# Generalization and discrimination tasks yield concordant measures of perceived distance between odours and their binary mixtures in larval *Drosophila*

**DOI:** 10.1242/jeb.100966

**Published:** 2014-06-15

**Authors:** Yi-chun Chen, Bertram Gerber

**Affiliations:** 1 Universität Würzburg, Biozentrum, Lehrstuhl für Genetik und Neurobiologie, 97074 Würzburg, Germany; 2 Otto von Guericke Universität Magdeburg, Institut für Biologie, Verhaltensgenetik, 39106 Magdeburg, Germany; 3 Center for Behavioral Brain Science (CBBS), 39106 Magdeburg, Germany; 4 Leibniz Institut für Neurobiologie (LIN), Abteilung Genetik von Lernen und Gedächtnis, 39118 Magdeburg, Germany

**Keywords:** *Drosophila melanogaster*, Memory, Olfaction, Perception, Generalization, Discrimination

## Abstract

Similarity between odours is notoriously difficult to measure. Widely used behavioural approaches in insect olfaction research are cross-adaptation, masking, as well as associative tasks based on olfactory learning and the subsequent testing for how specific the established memory is. A concern with such memory-based approaches is that the learning process required to establish an odour memory may alter the way the odour is processed, such that measures of perception taken at the test are distorted. The present study was therefore designed to see whether behavioural judgements of perceptual distance are different for two different memory-based tasks, namely generalization and discrimination. We used odour–reward learning in larval *Drosophila* as a study case. In order to challenge the larvae's olfactory system, we chose to work with binary mixtures and their elements (1-octanol, *n*-amyl acetate, 3-octanol, benzaldehyde and hexyl acetate). We determined the perceptual distance between each mixture and its elements, first in a generalization task, and then in a discrimination task. It turns out that scores of perceptual distance are correlated between both tasks. A re-analysis of published studies looking at element-to-element perceptual distances in larval reward learning and in adult punishment learning confirms this result. We therefore suggest that across a given set of olfactory stimuli, associative training does not grossly alter the pattern of perceptual distances.

## INTRODUCTION

Understanding perception is one of the more challenging tasks in science. Among many other difficulties, developing experimental handles on perception in non-verbal animals is a major practical concern. Widely used approaches in insect olfaction research are cross-adaptation (e.g. Cobb and Domain, 2000; Boyle and Cobb, 2005), masking (e.g. Kreher et al., 2008) and memory-based tasks (e.g. Smith, 1991; Guerrieri et al., 2005; Niewalda et al., 2011; Campbell et al., 2013; Parnas et al., 2013; Barth et al., 2014). In cross-adaptation, an odour A is presented at high concentration and for an extended period of time, such that responses are adapted out. If, in such an adapted state, animals still respond to a probe odour B, one can conclude that the two odours use at least partially non-overlapping input channels, and that they are discriminable. In turn, if, after adaptation to A, another probe odour A′ does not elicit a response, one can conclude that A and A′ share their input channels and thus should not be discriminable. For example, if you return your car to the garage after a faculty club dinner, you may be adapted to the smell of the heavy Havana cigars your dean likes to smoke. This adaptation may partially carry over to the smell of cigarettes but not to the smell of cat urine. Thus, you would hardly recognize that your rascal twin sons had been secretly smoking in the garage, while you would immediately realize that your neighbours' cat has paid another visit.

In masking tasks, a low-concentration probe odour B is presented in the background of a mask odour A that is at saturation concentration. If, under such conditions, the animals still respond to B, the two odours use at least partially non-overlapping processing streams, thus likewise arguing for the ability of the animals to discriminate them. In turn, if, in the background of odour A, the animals do not respond to another probe odour A′, the notion is that A and A′ share essential circuitry and are not discriminable. For example, after your faculty club dinner you may not recognize that in the background of your dean's third Havana your vice dean is lighting a Virginia cigar, while you would pick up on the smell of the coffee being served next door.

In associative, memory-based tasks, in principle two approaches are used: generalization and discrimination. In a generalization task, an odour A is rewarded but responses are tested for another, non-trained odour A′. To the extent that animals respond to A′ although it had never been rewarded, they regard A and A′ as similar. If, however, an odour B is tested after training with A and no responses are observed, the animals regard A and B as distinct. For example, the smell of Havana cigars may, after being a faculty member for some time, become associated with the peaceful comfort of the club, and this may partially carry over to the smell of Virginia cigars.

In a discrimination task, animals are differentially trained such that odour A is rewarded but B is not; after such training, the choice between both odours is tested. The rationale here is that such discrimination should be more difficult the more similar both odours are. For example, you may learn relatively quickly to tell apart the smell of Havana cigars from the smell of Virginia cigars if only one of them makes you sick, but it may take you much longer to tell apart the smell of two different kinds of Havana cigar.

However, a concern with memory-based approaches is that the learning process required for establishing an odour memory may alter the way the odour is processed, such that measures of perceived distance are distorted. For example, initially you may generalize between Havanas and Virginias and just lump them together as cigars, which are distinct in smell from cigarettes. However, with enough discriminative training, you can learn to tell Havanas and Virginias apart and regard their smell as hardly less distinct from each other than from cigarettes. Given this possibility for distortion in the relative judgements of similarity, the present study is designed to determine whether behavioural judgements of perceptual distance indeed are different for two different memory-based tasks. That is, do generalization and discrimination tasks yield correlated scores of perceptual distance?

We use odour–reward learning in larval *Drosophila melanogaster* as a study case (Scherer et al., 2003; Neuser et al., 2005; for reviews, see Gerber and Stocker, 2007; Gerber et al., 2009; Diegelmann et al., 2013). In order to challenge the larvae's olfactory system, we chose to work with binary mixtures and their elements. We determined the perceptual distance between each mixture and its elements first in a generalization task, and then in a discrimination task. This allowed us to ask whether the respectively determined scores of perceptual distance are concordant.

## RESULTS

### Experimental procedure

**Fig. 1.**
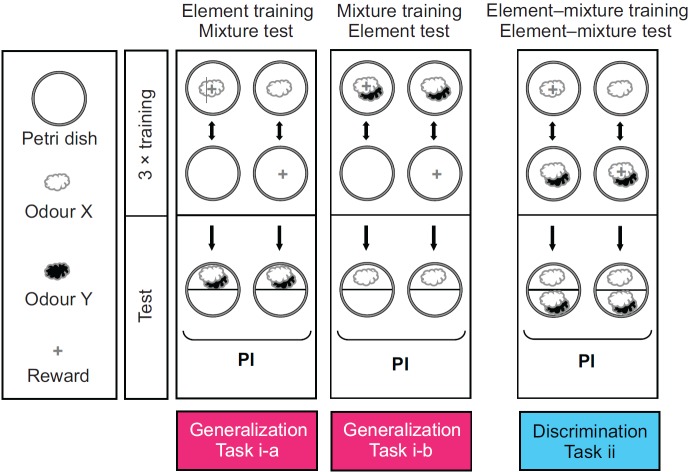
**General procedures of the generalization and discrimination tasks.** Task i is a generalization task. In Task i-a, larvae are trained to associate an odour X (open cloud) with a sugar reward, and are subsequently tested for their approach to a binary mixture containing the trained odour (XY; open and filled clouds); reciprocal groups are tested in the same way, yet after unpaired presentations of odour and reward. From the difference in preference between these two kinds of experimental group, the associative performance index (PI) is calculated. The same two-group design is used for Task i-b, except that animals are trained to associate a binary mixture with a sugar reward and are tested for their approach to one of its constituent elements. Task ii is a discrimination task, such that larvae are both trained and tested differentially between an odour versus a binary mixture containing it. The following odours were used, in all possible combinations, as X and Y (see Table 1 and Materials and methods for details): 1-octanol (1O), *n*-amyl acetate (AM), 3-octanol (3O), benzaldehyde (BA) and hexyl acetate (HA).

Using an odour element X and a binary mixture containing it (XY), we performed two types of task (Fig. 1): a generalization task (either as element-to-mixture generalization, or as mixture-to-element generalization), and a discrimination task [the following odours were used, in all possible combinations, as X and Y: 1-octanol (1O), *n*-amyl acetate (AM), 3-octanol (3O), benzaldehyde (BA) and hexyl acetate (HA); see Table 1 and Materials and methods for details].

### Task i-a

In an element-to-mixture generalization task, larvae were trained with an odour element (X) against a no-odour, ‘empty’ condition. Afterwards, they were tested for their preference for the trained element plus a previously non-trained odour (comprising a binary mixture XY). Thus, the larger the perceptual distance between X and XY, the less conditioned behaviour towards XY we should observe.

### Task i-b

In a corresponding mixture-to-element generalization task, larvae were trained with a binary mixture (XY) against the no-odour, ‘empty’ condition. Afterwards, they were tested for their preference for the contained element (X). Thus, again, the larger the perceptual distance between XY and X, the less conditioned behaviour towards X we should observe.

### Task ii

In the discrimination task, larvae were trained differentially between an odour element (X) against a binary mixture containing it (XY), and then were tested for their choice between these stimuli. Thus, in this task a larger perceptual distance between X and XY allows better discrimination and thus entails higher levels of conditioned behaviour.

The present experiments comprise 70 experimental groups, with a total sample size for the associative PI scores of *N*≈850 (as each PI score is based on the behaviour of approximately 60 larvae, *n*≈51,000).

### Task i: generalization

#### Learnability

**Fig. 2.**
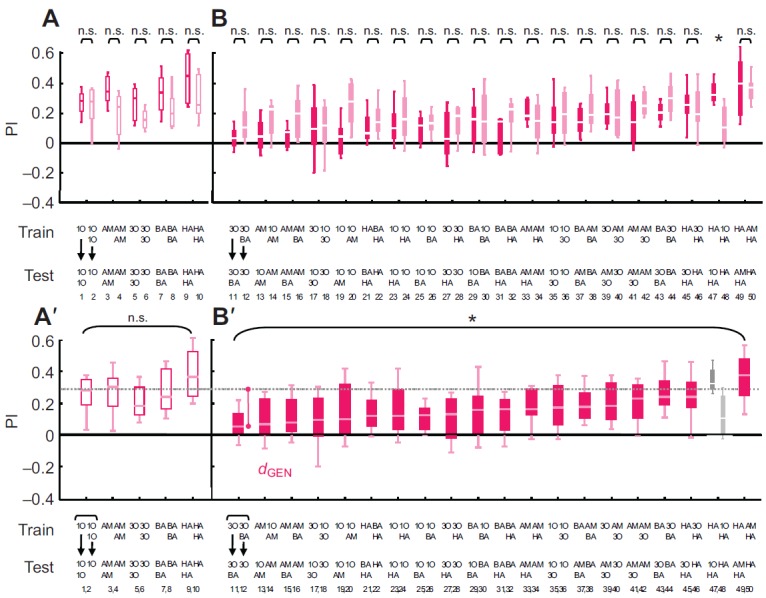
**Generalization.** Associative performance indices (PIs) are presented for the generalization task in which animals are trained with an element (X) and tested with a mixture containing it (XY), or vice versa. The numbers denoted at the bottom of each panel refer to the group numbers listed in Table 1. The odours used are listed below the figure. (A) Companion groups of larvae are trained with an element and tested with its double quantity, or vice versa. Performance indices are statistically indistinguishable in all cases (Mann–Whitney *U*-test: *P*>0.05/5, *N*=12 in all cases). (B) Companion groups of larvae are trained with an element and tested with a binary mixture containing it, or vice versa. Performance indices are equal in 19 of 20 of these cases (Mann–Whitney *U*-test: *P*>0.05/20 for Groups 11–46 and 49–50) except for 1O and 1O–HA (Group 47 versus 48; Mann–Whitney *U*-test: *P*<0.05/20). Sample sizes are 12 in all cases. (A′) Pooled scores of the companion groups from A. A comparison within the data set reveals statistically indistinguishable learnability across odours (Kruskal–Wallis test: *P*>0.05/2, *N*=24 in all cases), yielding a baseline of performance indices (dashed grey line represents the median of the pooled data). (B′) Pooled scores of the companion groups from B. The larger the perceptual distance between element and mixture, the less conditioned behaviour one should observe (i.e. the smaller the PI scores should be). Thus, in this task perceptual distance can be approximated by the *d*_GEN_ scores, measuring how much smaller PI scores are relative to baseline. A Kruskal–Wallis test (*P*<0.05/2, *N*=24 in all cases) reveals that depending on the odours used, perceived distance differs between elements and mixture. Note that because no unambiguous measure of element–mixture similarity could be obtained for the case of 1O and 1O–HA (see B), this stimulus pair cannot be included in this Kruskal–Wallis test. See Fig. 1 legend for odour definitions.

We first needed to test whether the chosen odour concentrations allow for fair tests of generalization. We found that twofold increases versus twofold decreases in odour concentration between training and testing did not lead to statistically distinguishable asymmetries in associative performance indices (Mann–Whitney *U*-tests: from left to right, *U*=68, 33, 35, 40, 39, *P*>0.05/5, *N*=12 in all five cases; Fig. 2A). This is consistent with Mishra et al. (Mishra et al., 2013), who reported that 10- to 100-fold changes in odour intensity are required to compromise recognition of the trained stimulus during the test. In any event, the current result allowed reasonably fair comparisons of conditioned behaviour towards a mixture XY after element training with X (featuring a twofold increase in total odour concentration) versus the case in which behaviour towards X was tested after training with XY (featuring a twofold decrease in total odour concentration). Furthermore, as such symmetry was found for all five odours, we could pool these respective pairs of groups to compare learnability between odours. It turned out that odour identity did not have a significant effect on conditioned behaviour (Kruskal–Wallis test: *H*=10.42, d.f.=4, *P*>0.05/2, *N*=24 in all cases Fig. 2A′). Therefore, we could approximate a baseline level of conditioned behaviour for the case when the trained stimulus was indeed presented at testing (either as a doubled total amount of odour, or when reduced by half), such that levels of generalization could be approximated against this baseline (dashed line in Fig. 2A′,B′).

**Table 1.**
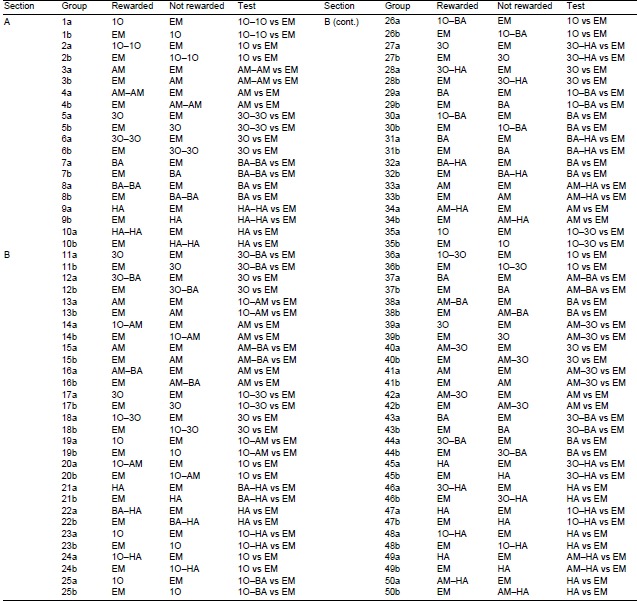
Summary of experimental treatments for training and testing in Task i

#### Generalization between element and mixture is symmetrical and differs across element–mixture pairs

Generalization between element and mixture (Fig. 1, Task i) was typically symmetrical: considering groups 49 and 50 as example, the same level of conditioned behaviour to the mixture AM–HA was observed after training with HA (Fig. 2B, group 49) as when the AM–HA mixture was trained and the HA element was tested (Fig. 2B, group 50). Such symmetry was found in 19 of 20 cases (Mann–Whitney *U*-tests: *U*=25 to 72, *P*>0.05/20, *N*=12 in all cases; Fig. 2B) [the exception was the case of HA and 1O–HA (group 47 versus 48; Mann–Whitney *U*-test: *U*=19, *P*<0.05/20, *N*=12, 12), such that for HA and 1O–HA no unambiguous measure of perceived distance could be obtained]. Pooling the data for element-to-mixture generalization with the data for mixture-to-element generalization could thus yield an approximation of perceptual distance (denoted as *d*_GEN_ in Fig. 2B′) between element and mixture. For example, as

very little generalization was found between 3O and the 3O–BA mixture (Fig. 2B′, groups 11, 12), the perceptual distance between 3O and the 3O–BA mixture is large.

Perceived distances differed between element–mixture pairs (Kruskal–Wallis test: *H*=63.77, d.f.=18, *P*<0.05/2; *N*=24 in all cases; Fig. 2B′) [note that the case of HA and 1O–HA (groups 47 and 48) was not included in this analysis, because for this case no unambiguous measure of element–mixture similarity could be obtained], prompting the question of whether the found perceived distances are indeed the property of the employed elements and mixtures, or whether they reflect properties of the employed task. We therefore used a discrimination task to determine whether these measures of perceived distance are correlated across tasks.

### Task ii: discrimination

**Fig. 3.**
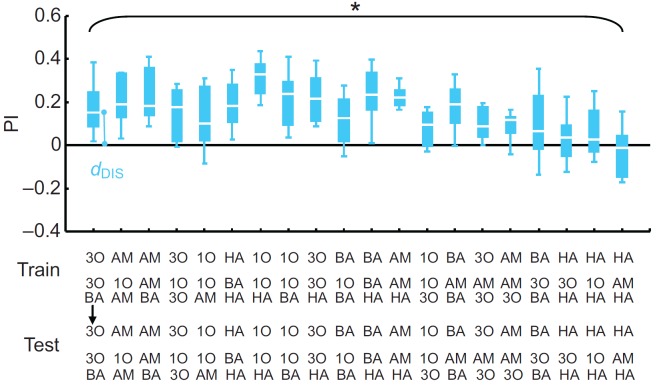
**Discrimination.** Associative performance indices (PIs) are presented for the discrimination task, in which animals were trained and tested differentially with any one of the odour elements (X) versus a mixture containing it (XY). The larger the perceptual distance between element and mixture, the easier should discrimination be and thus the more conditioned behaviour one should observe (i.e. the higher the PI scores should be). Thus, in this task perceptual distance can be approximated by the *d*_DIS_ scores. A Kruskal–Wallis test (*P*<0.05, *N*=12 in all cases) reveals that depending on the odours used, perceived distance differs between elements and mixture. See Fig. 1 legend for odour definitions.

**Fig. 4.**
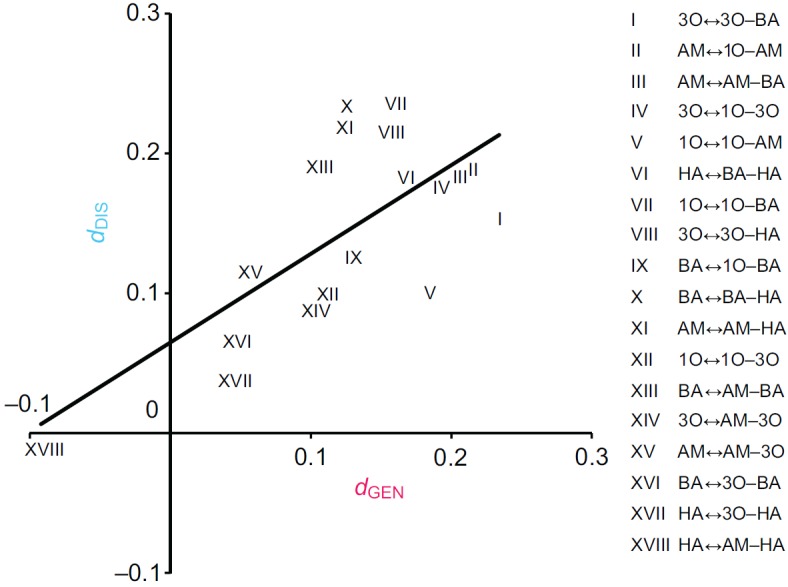
**Correlation of perceptual distances derived from generalization and discrimination tasks.** Measures of element–mixture perceptual distances correlate between generalization and discrimination tasks. The plot presents *d*_DIS_ on the *y*-axis and *d*_GEN_ on the *x*-axis (Spearman's rank correlation: *r*_S_=0.52, *P*<0.05, *N*=18). See Fig. 1 legend for odour definitions.

Larvae were trained to discriminate between any one of the odour elements (X) versus a mixture containing it (XY); at test, the larvae were then offered the choice between these very two trained stimuli (Fig. 1, Task ii). If the perceived distance is large, such discrimination should be easy and scores should be correspondingly high. Thus, we depicted discrimination-based perceived distance between element and mixture as *d*_DIS_ in Fig. 3. Levels of discrimination did differ across element–mixture combinations (Kruskal–Wallis test: *H*=60.05, d.f.=19, *P*<0.05, *N*=12 in all cases; Fig. 3), such that we could ask whether the measures of element–mixture perceptual distances correspond for the present discrimination and the above generalization task. Towards this end, we plotted – for each element and the mixtures containing it – the *d*_GEN_ value against the *d*_DIS_ value (Fig. 4). It turned out that the element–mixture perceptual distances are correlated for the generalization and the discrimination task (Spearman's rank correlation: *r*_S_=0.52, *P*<0.05, *N*=18; Fig. 4).

## DISCUSSION

**Fig. 5.**
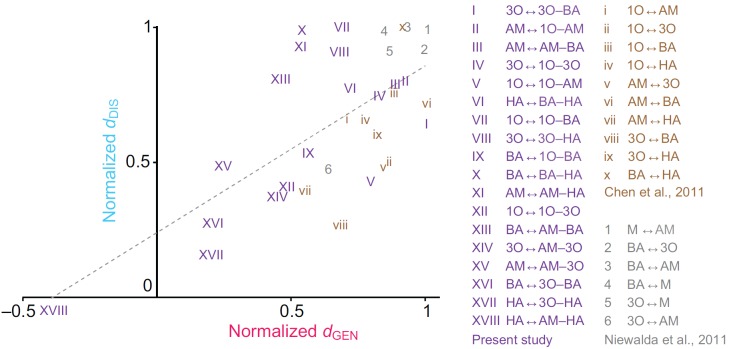
**Correlation of normalized perceptual distance scores from this study and the literature.** Normalized perceptual distances correlate between generalization and discrimination tasks not only for the present study (I–XVIII, purple), but also for previously published ones probing for element–element perceptual distances using a larval odour–sugar learning paradigm [i–x, brown (Chen et al., 2011)] and for element–element distances using odour–shock learning in adult *Drosophila* [1–6, grey (Niewalda et al., 2011)]. The plot presents normalized *d*_DIS_ scores on the *y*-axis and normalized *d*_GEN_ scores on the *x*-axis (Spearman's rank correlation: *r*_S_=0.53, *P*<0.05, *N*=34) (the stimulus combinations and odours used in the respective studies are listed in the keys). Thus, across all three studies, distances scores approximated from generalization and discrimination tasks were concordant. M, 4-methylcyclohexanol. See Fig. 1 legend for other odour definitions.

This study was specifically designed to determine whether the type of mnemonic task influences measures of element–mixture perceptual distance. This is not the case: perceived distance scores from a generalization and a discrimination task strongly correlate (Fig. 4). This is confirmed when reconsidering normalized published *d*_GEN_ and *d*_DIS_ scores for element–element perceptual distances from larval reward learning (Chen et al., 2011) and adult punishment learning (Niewalda et al., 2011; see also Campbell et al., 2013) (Spearman's rank correlation: *r*_S_=0.53, *P*<0.05, *N*=34; Fig. 5). Whether such task-independence extends to tasks that do not use associative learning but,

for example, masking or cross-adaptation to measure perceived distance (see Introduction) remains to be investigated.

Of note, the correlation plot in Fig. 5 intersects the *y*-axis at approximately 0.25. If taken at face value, this implies cases where there is full generalization (*d*_GEN_=0), but discrimination is still possible (*d*_DIS_>0). Indeed, for 3-octanol and 1-octen-3-ol this is what we had previously found (Mishra et al., 2010). Clearly, successful performance after discrimination training requires that there is at least some initial difference in processing between the two odours before training. This difference can either be ignored, leading to full generalization, or it can be enhanced, leading to successful discrimination (Mishra et al., 2010) [see the recent paper by Barth et al. (Barth et al., 2014) pointing to the so-called mushroom body gamma lobes as a site of such enhancement]. In other words, the notion that the correlation plot in Fig. 5 intersects the *y*-axis at approximately 0.25 is consistent with an enhancement of perceived distance by discrimination training. Such enhancement, however, does not distort the patterns of perceived distance across a set of odours (Figs 4, 5): as a rule, when there is relatively strong generalization between two olfactory stimuli, they are relatively hard to discriminate. In this sense, the type of associative task is not a major determinant for measures of perceptual distance.

## MATERIALS AND METHODS

### General methods

General methods (and experimental procedures; see below) follow Chen et al. (Chen et al., 2011), Mishra (Mishra, 2011), Chen (Chen, 2012) and Mishra et al. (Mishra et al., 2013) [see also Gerber et al. (Gerber et al., 2013) for a hands-on manual]. Third-instar larvae of the *Drosophila melanogaster* wild-type strain Canton-S, in their feeding stage (5 days after egg laying), were used throughout. Animals were kept in standard-medium mass culture (25°C, 60–70% relative humidity, 14 h:10 h light:dark cycle). Experiments were performed under a fume hood at room temperature (21–26°C).

Olfactory stimuli were prepared by putting 10 μl of odorant into Teflon containers of 5 mm diameter that could be closed by a lid perforated with seven holes, each of 0.5 mm diameter. The set of used olfactory stimuli included 1-octanol (1O; Sigma-Aldrich, CAS 111-87-5), *n*-amyl acetate (AM; Merck, CAS 628-63-7), 3-octanol (3O; Merck, CAS 589-98-0), benzaldehyde (BA; Fluka, CAS 100-52-7), hexyl acetate (HA; Sigma-Aldrich, CAS 142-92-7) and no odour, which is a container without any odorant (empty: EM). Odorants were diluted in paraffin oil (1O: 1:100; AM: 1:3333; 3-O: 1:10^5^; BA: 1:100; HA: 1:100; paraffin oil: CAS 8012-95-1; Merck, Darmstadt, Germany), which is without behavioural significance in the present type of paradigm (Saumweber et al., 2011). At these dilutions, learnability was equal for all odours, and at the lower limit of the asymptote of learning performance (Mishra et al., 2013). To present binary mixtures, two containers were used, one for each element at the respective dilution mentioned above (Eschbach et al., 2011).

Petri dishes (85 mm diameter, Sarstedt, Nümbrecht, Germany) were filled with only agarose (1%, electrophoresis grade, CAS 9012-36-6) or with agarose additionally containing sugar as reward (2 mol l^−1^ fructose, CAS: 57-48-7; both from Roth, Karlsruhe, Germany). Upon solidification, the Petri dishes were covered with their lids, left at room temperature and used the next day.

### General experimental procedures of the learning experiments

First, we replaced the Petri dish lids with lids perforated by 15 centrally located 1 mm holes for better aeration. A spatula of medium with larvae was taken from the culture vial in order to collect a group of approximately 30 animals; these were gently rinsed in water immediately before being used for the experiment. In all experiments, the larvae underwent one of two reciprocal regimens (Fig. 1). Either they received training such that odour A was paired with reward (+) while odour B was not (A+ // B; the // symbol denotes that A+ and B were presented in separate trials), or they received reciprocal training (A // B+) (the chemical identity of A and B is specified in the Results and in Table 1). Afterwards, the larvae were offered a choice between A versus B in the test. Importantly, animals from both training regimens thus undergo the same handling and equivalent exposure to odours and reward; what differs between them is specifically the odour–reward contingency [please note that half of the cases featured A as the first stimulus (A+ // B and A // B+), whereas in the other cases B was first (B // A+ and B+ // A)]. Associative memory thus is shown specifically by a difference in test behaviour between these reciprocally trained sets of larvae.

This associative difference is quantified by the performance index (PI; see below).

For training, two containers were placed on opposite sides of the Petri dish, 7 mm from the edge, both loaded with the same odour stimulus. The Petri dish contained the sugar reward or not, depending on group assignment (see Table 1). Animals were placed onto the Petri dish within a central 7 mm strip and the lid was closed. Five minutes later, they were placed onto a fresh Petri dish with the respective other odour–tastant combination. This cycle was repeated two more times. Then, the choice of the animals between two test stimuli was determined. To this end, one odour container was placed on either side of the test Petri dish not containing the sugar reward, each equipped with a different stimulus to generate a choice situation. For example (for the deviations, see the description of the tasks below, as well as the Results and Table 1), containers were loaded with odour A on one side and odour B on the other side (Test A — B). Then the larvae were placed on the central strip. The number of animals on the A side, the B side, the central strip and those that had wandered off onto the lid was determined after 3 min. This allowed us to calculate a preference score (PREF) by subtracting the number of animals on the B side from those on the A side (@B and @A, respectively), divided by the total:
(1)
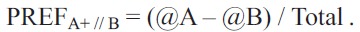



Then, another group of 30 animals was trained in a reciprocal manner, and the PREF score was determined in the same way:
(2)
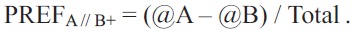



To determine whether preferences associatively depended on training regimen, we calculated a PI from these two reciprocally trained groups ranging from −1 to 1 as:
(3)
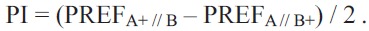



Positive PIs thus indicate conditioned approach; negative PIs represent conditioned avoidance. Data from experimental conditions to be compared statistically were obtained in parallel. Larvae were trained and tested only once. Please recall that either an odour element or a mixture containing it (denoted as X and XY in the following) could be defined as A or B.

### Specific features of the learning tasks

A series of element–mixture tasks was performed (for a full list of all experimental groups, see Table 1). In the following, X always denotes an odour element, and XY a binary mixture containing it. First, we wanted to test for generalization between X and XY, such that either X was trained and XY was tested, or XY was trained and X was tested. We decided to use the same amount of X in training and in the test, i.e. when it was presented as an element and when presented within the XY mixture. Consequently, the total amount of odour was higher in the mixture. We therefore initially tested whether doubling the amount of odour between training and test (odd-numbered groups in Table 1A), or respectively reducing it by half (even-numbered groups in Table 1A), would introduce any asymmetry in test behaviour. As this was not the case (see Results and Fig. 2A), we pooled these groups and compared the learnability between odours, which revealed no difference (see Results and Fig. 2A′). This allowed for a reasonable approximation of a baseline level of conditioned behaviour when the trained odour was presented at test (either in the context of a doubled total amount of odour, or with a total amount of odour reduced by half), relative to which levels of generalization could be judged.

### Task i-a: element-to-mixture generalization

In an element-to-mixture generalization task (odd-numbered groups in Table 1B), larvae were trained with any one of the five odour stimuli (element X) against EM. Afterwards, they were tested for their choice between the trained element plus any one of the four remaining non-trained odours (which we labelled Y, together comprising a binary mixture XY) versus EM. An abbreviated form for this generalization task may thus read as: Train: X // EM and Test: XY — EM.

Thus, the larger the perceptual distance between X and XY, the less conditioned behaviour towards XY we should observe (i.e. the smaller the PI scores should be).

### Task i-b: mixture-to-element generalization

In a corresponding mixture-to-element generalization task (even-numbered groups in Table 1B), larvae were trained with any of the 25 possible binary mixtures (XY) against EM. Afterwards, they were tested for their choice between either of the involved elements (X) versus EM: Train: XY // EM and Test: X — EM.

Again, the larger the perceptual distance between XY and X, the less conditioned behaviour towards X we should observe (i.e. the smaller the PI scores should be).

### Task ii: discrimination

In an element versus mixture discrimination task, larvae were trained differentially between any of the five elements (X) against a binary mixture containing it (XY), and then were tested for their choice between these stimuli: Train: X // XY and Test: X — XY.

Thus, in this task, a larger perceptual distance between X and XY entails more conditioned behaviour (i.e. a larger PI score).

### Data display and statistics

Data are displayed as box plots, showing the median as a bold line and the 25/75% and 10/90% quantiles as box boundaries and whiskers, respectively.

Non-parametric analyses were employed throughout (Kruskal–Wallis tests for comparisons across multiple-groups, Mann–Whitney *U*-tests for two-group comparisons), using Statistica (Statsoft, Hamburg, Germany). Significance is inferred if *P*<0.05. If, within one analysis, multiple tests were performed, the significance level was corrected by dividing it by the number of comparisons (Bonferroni correction); this ensures that the total error rate of the analysis remains below 5%. For example, if three such comparisons were made, *P*<0.05/3 was used as the significance level.

Experimenters were blind with respect to the reward status of the Petri dishes.

## Supplementary Material

Supplementary Material
