# Supplementary material for: Generalization and discrimination tasks yield concordant measures of perceived distance between odours and their binary mixtures in larval Drosophila
Source: J Exp Biol. 2014 Jun 15;217(12):2071–7. doi: 10.1242/jeb.100966 (PMC4191342; doi:10.1242/jeb.100966)
Supplement: Supplementary Material [file supp_217_12_2071__index.html]

Generalization and discrimination tasks yield concordant measures of perceived distance between odours and their binary mixtures in larval Drosophila — Supplementary Material 

# Generalization and discrimination tasks yield concordant measures of perceived distance between odours and their binary mixtures in larval *Drosophila*

## JEB100966 Supplementary Material

**Files in this Data Supplement:**

- **Supplementary Material**
