# Supplementary material for: Generalization and discrimination tasks yield concordant measures of perceived distance between odours and their binary mixtures in larval Drosophila
Source: J Exp Biol. 2014 Jun 15;217(12):2071–7. doi: 10.1242/jeb.100966 (PMC4191342; doi:10.1242/jeb.100966)
Supplement: Supplementary Material [file supp_217.12.2071_JEB100966.pdf]

Chen & Gerber, JEB 2014 Supplementary material

Figure legends

Figure S1 Preference scores, task i

Preference scores of the reciprocally trained groups of larvae from which the associative Performance Indices presented in Fig.s 2A, B were derived. Preference scores in (A) correspond to the Performance Indices from Fig. 2A. Reciprocal groups received either paired or unpaired presentation of odour and reward (e.g. for the first and second plot: 1O+ // EM training, or 1O // EM+ training; testing was then carried out as 1O1O – EM) (recall EM denotes an empty odour container). Companion groups of larvae were trained with an element and tested with its double quantity, or vice versa (e.g. for the first and third plot: train 1O+ // EM, test 1O1O -- EM, or train 1O1O+ // EM, test 1O -- EM). Preference scores in (B) likewise correspond to the Performance Indices from Fig. 2B.

Figure S2 Preference scores, task ii

Preference scores of the reciprocally trained groups of larvae underlying the associative Performance Indices presented in Fig. 3.

Figure S1A

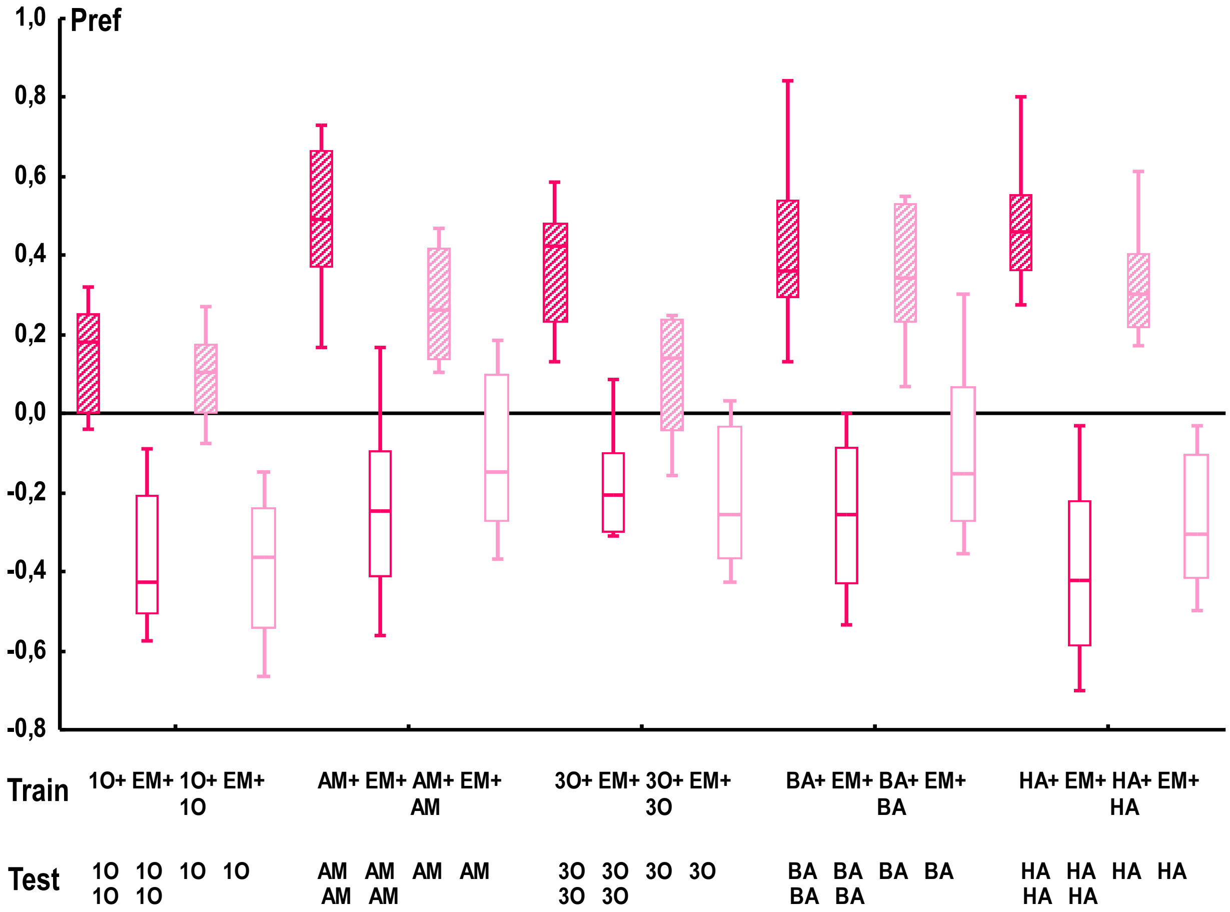

Pref
1,0
0,8
0,6
0,4
0,2
0,0
-0,2
-0,4
-0,6
-0,8
Train
1O+ EM+ 1O+ EM+
1O
AM+ EM+ AM+ EM+
AM
3O+ EM+ 3O+ EM+
3O
BA+ EM+ BA+ EM+
BA
HA+ EM+ HA+ EM+
HA
Test
1O 1O 1O 1O
1O 1O
AM AM AM AM
AM AM
3O 3O 3O 3O
3O 3O
BA BA BA BA
BA BA
HA HA HA HA
HA HA

Figure S1B

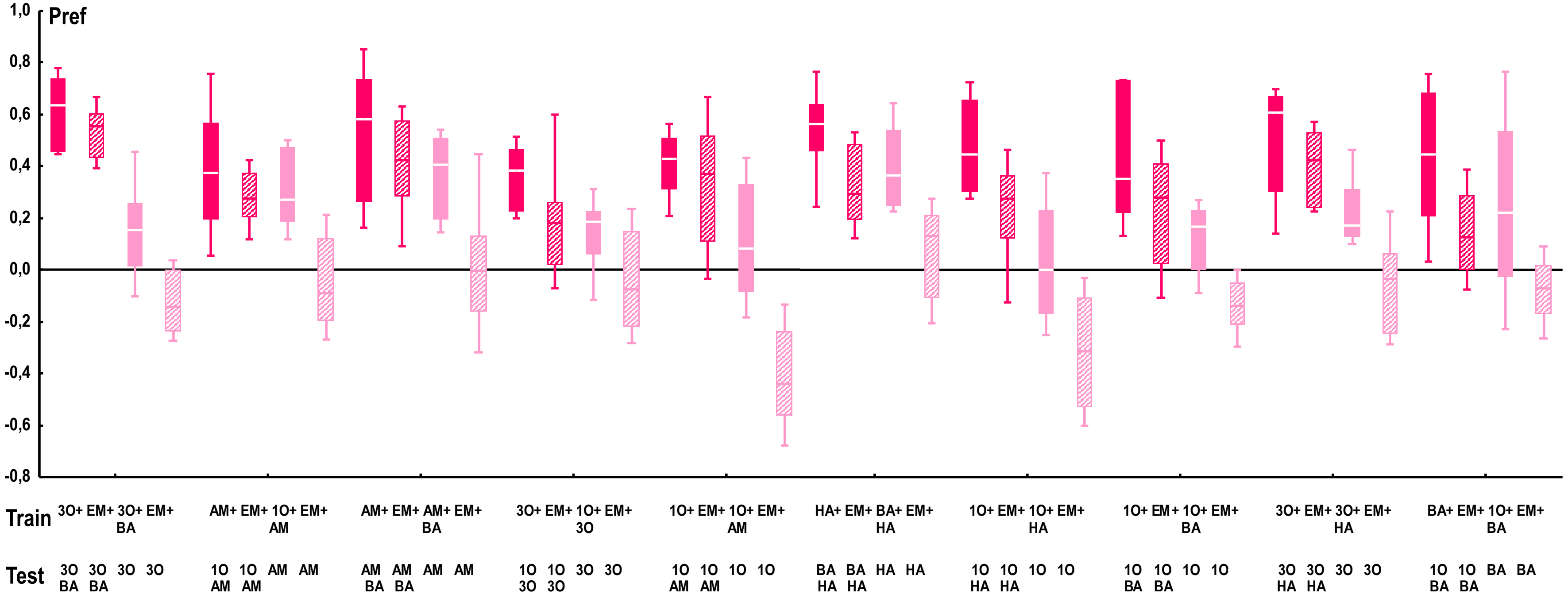

ctd.

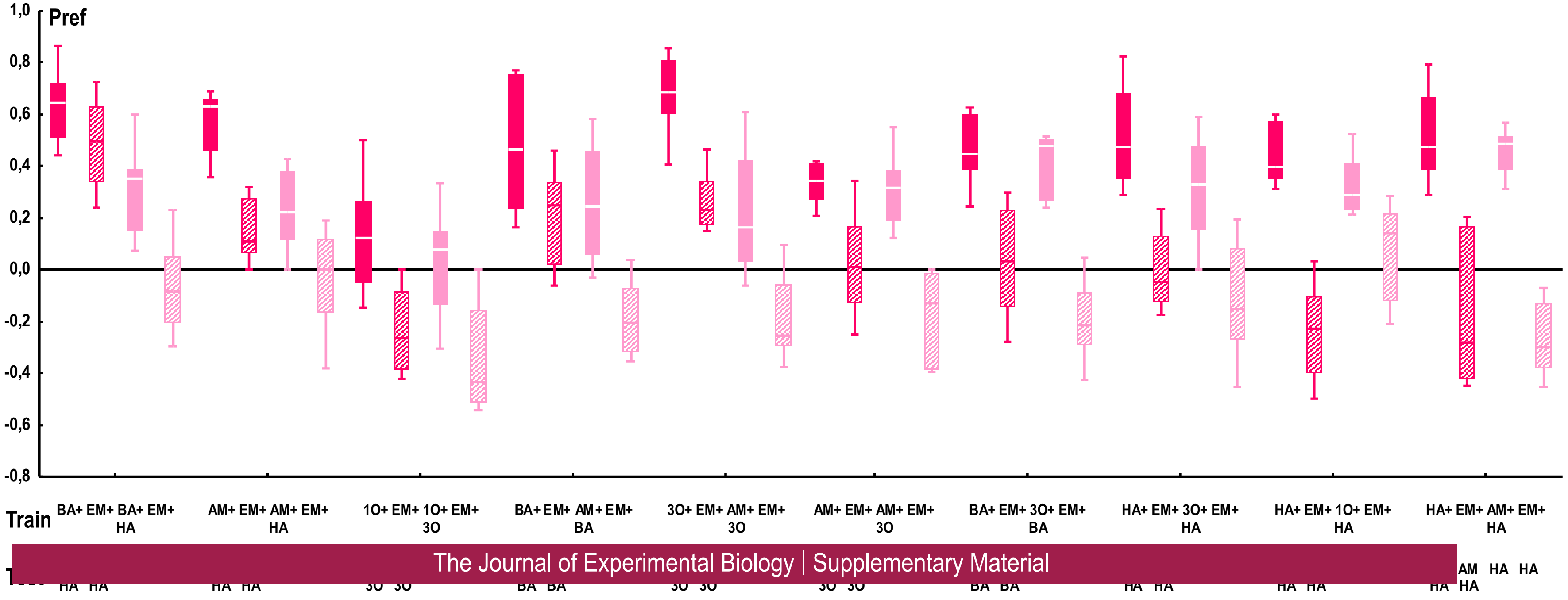

Figure S2

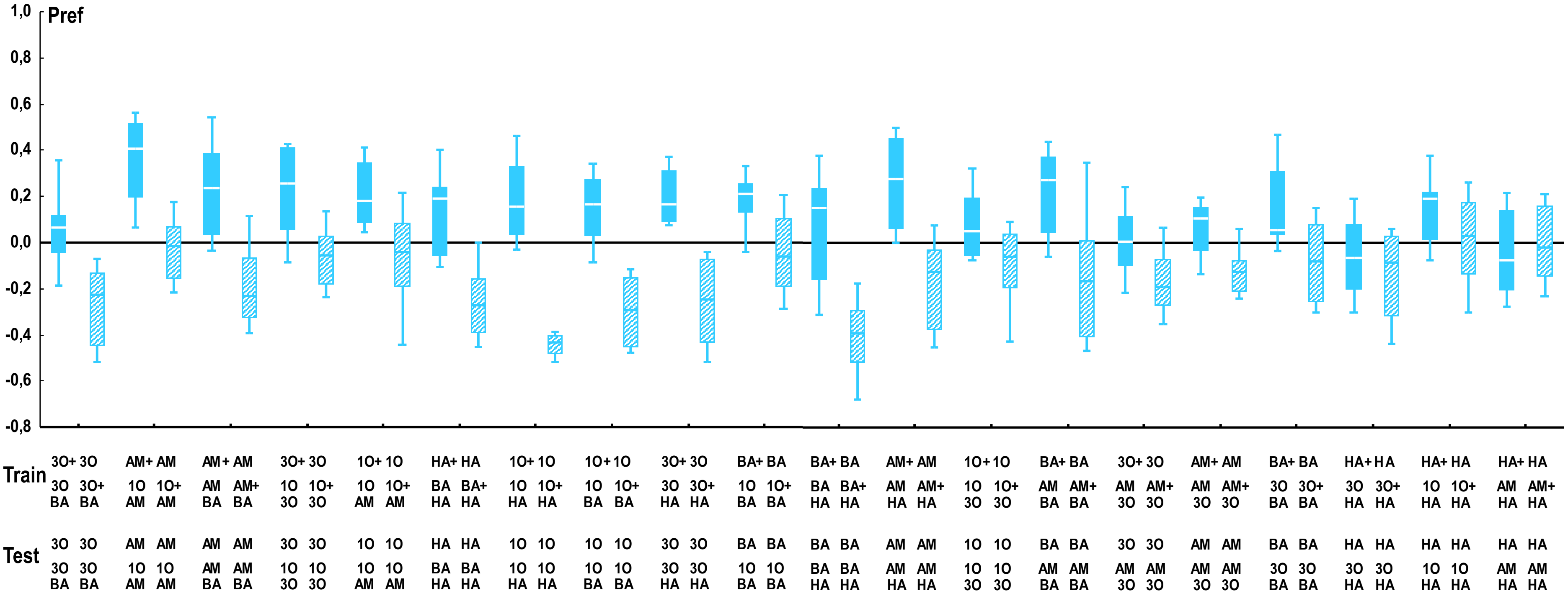
Pref
1,0
0,8
0,6
0,4
0,2
0,0
-0,2
-0,4
-0,6
-0,8
Train
3O+ 3O
3O 3O+
BA BA
AM+ AM
1O 1O+
AM AM
AM+ AM
AM AM+
BA BA
3O+ 3O
1O 1O+
3O 3O
1O+ 1O
1O 1O+
AM AM
HA+ HA
BA BA+
HA HA
1O+ 1O
1O 1O+
HA HA
1O+ 1O
1O 1O+
BA BA
3O+ 3O
3O 3O+
HA HA
BA+ BA
1O 1O+
BA BA
BA+ BA
BA BA+
HA HA
AM+ AM
AM AM+
HA HA
1O+ 1O
1O 1O+
3O 3O
BA+ BA
AM AM+
BA BA
3O+ 3O
AM AM+
3O 3O
AM+ AM
AM AM+
3O 3O
BA+ BA
3O 3O+
BA BA
HA+ HA
3O 3O+
HA HA
HA+ HA
1O 1O+
HA HA
HA+ HA
AM AM+
HA HA
Test
3O 3O
3O 3O
BA BA
AM AM
1O 1O
AM AM
AM AM
AM AM
BA BA
3O 3O
1O 1O
3O 3O
1O 1O
1O 1O
AM AM
HA HA
BA BA
HA HA
1O 1O
1O 1O
HA HA
1O 1O
1O 1O
BA BA
3O 3O
3O 3O
HA HA
BA BA
1O 1O
BA BA
BA BA
BA BA
HA HA
AM AM
AM AM
HA HA
1O 1O
1O 1O
3O 3O
BA BA
AM AM
BA BA
3O 3O
AM AM
3O 3O
AM AM
AM AM
3O 3O
BA BA
3O 3O
BA BA
HA HA
3O 3O
HA HA
HA HA
1O 1O
HA HA
HA HA
AM AM
HA HA
